# Association between dietary thiamin, riboflavin and niacin intake and hypertension and increased pulse pressure: A cross-sectional study of the National Health and Nutrition Examination Survey 1999–2023

**DOI:** 10.1097/MD.0000000000049149

**Published:** 2026-06-12

**Authors:** Siqi Ma, Qu Jin, Keying Yu, Hezeng Dong, Yanmei Lu, Liping Chang

**Affiliations:** a Department of Cardiology, Changchun University of Traditional Chinese Medicine, Changchun, China; b Cardiology Department, Changchun University of Traditional Chinese Medicine Affiliated Hospital, Changchun, China.

**Keywords:** hypertension, NHANES, niacin, pulse pressure, riboflavin, thiamin

## Abstract

This cross-sectional study aimed to examine the associations between total intake of 3 B vitamins (thiamin, riboflavin, niacin) and hypertension and increased pulse pressure in U.S. adults, and to explore potential nonlinear relationships.Data from the U.S. National Health and Nutrition Examination Survey (NHANES) 1999–2023 were analyzed. A total of 17,484 participants aged ≥ 20 years were included. Total vitamin intake (diet plus supplements) was categorized into quartiles. Hypertension was defined as SBP ≥ 140 mm Hg or DBP ≥ 90 mm Hg; increased pulse pressure as PP ≥ 60 mm Hg. Multivariable logistic regression and restricted cubic spline (RCS) models were used, adjusting for demographic, socioeconomic, and clinical factors.In fully adjusted models, the highest quartile of total thiamin (OR 1.17, 95% CI 1.04–1.31), riboflavin (OR 1.23, 95% CI 1.10–1.39), and niacin (OR 1.16, 95% CI 1.03–1.30) intake was positively associated with hypertension (P-trend < 0.05 for all). Conversely, only riboflavin was inversely associated with increased pulse pressure (OR 0.84, 95% CI 0.75–0.94). RCS analysis revealed a J-shaped nonlinear association between niacin intake and hypertension (P-nonlinear = 0.041), with the lowest risk observed at 30–40 mg/day. A suggestive inflection point was identified at 46.163 mg/day (*P* = .087). No clear thresholds were found for thiamin or riboflavin. Subgroup analyses showed largely consistent associations.This study suggests that high total intake of B vitamins may be associated with higher odds of hypertension, while riboflavin may be inversely associated with pulse pressure. The findings highlight the importance of considering total nutrient intake and caution against excessive supplementation. However, as a cross-sectional analysis, causality cannot be inferred, and residual confounding cannot be ruled out.

## 1. Introduction

Hypertension affects over 1 billion adults worldwide and is a major contributor to significant health challenges within global public health systems, with its prevalence increasing year on year.^[1,2]^ In the United States, hypertension affects 48.1% of adults – an estimated 119.9 million individuals.^[3]^ Hypertension is a leading risk factor for ischemic heart disease, stroke, chronic kidney disease, and other cardiovascular disorders, and it remains a principal driver of premature mortality.^[4,5]^ Blood-pressure reduction demonstrably lowers the incidence of multiple diseases, yet global control remains poor: fewer than 20% of hypertensive patients achieve target levels.^[6]^ Hence, pinpointing modifiable determinants and preventing hypertension to curtail cardiovascular risk and mortality is a global health imperative.

Hypertension and elevated pulse pressure arise from the interplay of dietary, behavioral, genetic, physiological, socioeconomic, and environmental factors.^[7,8]^ Diet is a key modifiable determinant of chronic disease prevention, including hypertension, cardiovascular disease, cancer, and type 2 diabetes.^[9,10]^ Accumulating evidence indicates that discrete dietary patterns and single nutrients can both precipitate and intensify hypertension and elevated pulse pressure, thereby delaying achievement of sustained disease remission.^[11]^ Recent analyses indicate that high carbohydrate intake correlates with elevated systolic blood pressure, but shows no association with isolated systolic, isolated diastolic, or combined systolic–diastolic hypertension phenotypes.^[12]^ Acute coffee ingestion transiently elevates blood pressure; however, meta-analytic evidence shows no incremental long-term hypertension risk.^[13]^ A cross-sectional analysis reported inverse associations between hypertension prevalence and aggregate intake of unsaturated, omega-3 and omega-6 fatty acids, including fish oil, α-linolenic, linoleic, and arachidonic acids.^[14]^ A separate investigation demonstrated a robust positive association between hypertension and dietary saturated fatty acids – specifically tetradecanoic (C14:0), hexadecanoic (C16:0), and octadecanoic (C18:0) acids.^[15]^ In obese participants receiving antihypertensive therapy, greater riboflavin intake was associated with attenuated increments in systolic and pulse pressure.^[8]^

B vitamins, including thiamin (B1), riboflavin (B2), and niacin (B3), are water-soluble micronutrients involved in fundamental cellular processes such as energy metabolism, redox reactions, and 1-carbon metabolism. These pathways are implicated in endothelial function, vascular tone regulation, and oxidative stress – key mechanisms underlying blood pressure homeostasis. For instance, thiamin acts as a cofactor for enzymes in carbohydrate metabolism and may influence endothelial nitric oxide production.^[16–18]^ Riboflavin is a precursor for flavin coenzymes (FAD, FMN) crucial for redox balance and homocysteine metabolism, the latter being an independent risk factor for vascular dysfunction.Niacin participates in NAD(P)H synthesis, affecting nitric oxide bioavailability and prostaglandin-mediated vasodilation.Although population-based studies have linked thiamin, riboflavin and niacin intake to cardiovascular health, their specific impact on adults with established hypertension or elevated pulse pressure remains under-examined.Furthermore, existing evidence is inconsistent, with some studies suggesting protective effects and others showing null or even adverse associations, possibly due to differences in study design, population, assessment of intake, and adjustment for confounders.Therefore, this study provides a comprehensive evaluation of total vitamin B intake in relation to hypertension and elevated pulse pressure in a large, nationally representative U.S. cohort.

## 2. Materials and methods

### 2.1. Study population

This cross-sectional analysis used 1999–2023 cycles of the National Health and Nutrition Examination Survey (NHANES). The Centers for Disease Control and Prevention collected, compiled, and deposited the data in the National Center for Health Statistics (NCHS) repository; methodological details are publicly available (https://wwwn.cdc.gov/nchs/nhanes/AnalyticGuidelines.aspx). The NCHS Research Ethics Review Board approved all protocols, and participants provided written informed consent; secondary analyses of de-identified data do not require additional institutional review.

**Figure 1.**
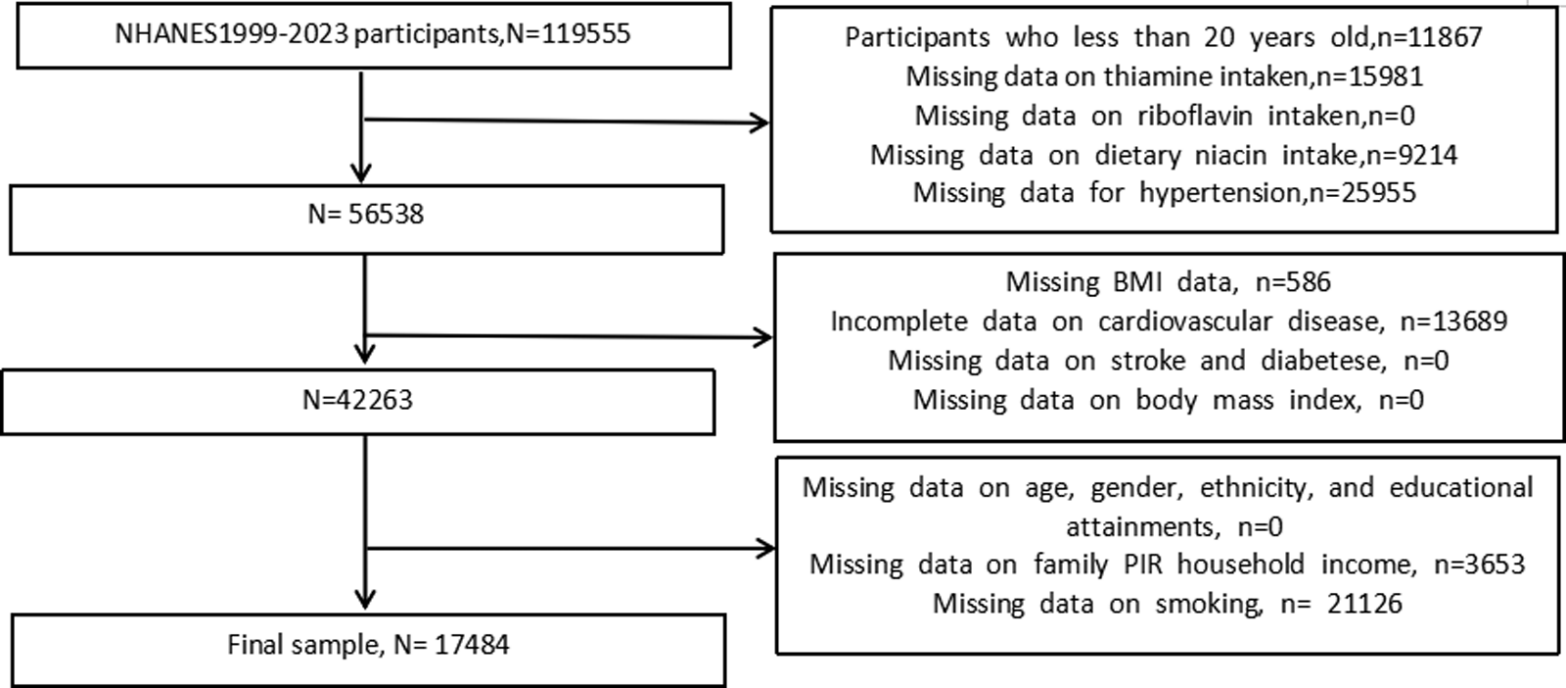
Flow chart of study participants. N = total number of participants, n = number excluded.

We excluded individuals aged < 20 y and those lacking data on B-vitamin intake, blood pressure variables, or covariates.Specifically, among the initial 119,555 respondents across 12 NHANES cycles, we sequentially excluded: participants aged < 20 years (n = 52,110), those with missing data on B-vitamin intake (n = 38,752), missing blood pressure measurements (n = 9623), missing data on body mass index (n = 586), and missing data on other key covariates (n = 1000). After these exclusions, 17,484 adults remained for the final analysis. The detailed flow is presented in Figure 1.

### 2.2. Variables

#### 2.2.1. Nutrient intake

We restricted micronutrient exposure to water-soluble B vitamins – thiamin (B1), riboflavin (B2) and niacin (B3). Dietary intake was assessed using the first 24-hour dietary recall interview. Energy and nutrient densities of all reported foods were obtained from the Food and Nutrient Database for Dietary Studies (FNDDS) corresponding to each NHANES cycle. Vitamin intakes were computed from actual consumption weights.During the Mobile Examination Center (MEC) visit, participants recalled all vitamin/mineral supplements used during the previous 30 days. For each product, daily supplemental intake (mg d^−1^) was derived as [(frequency × unit dose × quantity per time)/ 30]. Dietary and supplemental values were summed to yield total daily intake, and individuals were classified into quartiles (Q1–Q4) for subsequent dose–response analyses.

#### 2.2.2. Hypertension and increased pulse pressure

Based on the pathogenesis of hypertension, individuals with hypertension are defined as having an average systolic blood pressure (SBP) of ≥ 140 mm Hg or an average diastolic blood pressure (DBP) of ≥ 90 mm Hg.^[19,20]^ Blood pressure was measured by trained examiners using a standardized protocol. Three to four consecutive readings were obtained after a 5-minute rest. For this analysis, the average of all available readings was used. A large pulse pressure (PP) is defined as ≥ 60 mm Hg, calculated by subtracting the mean diastolic pressure from the mean systolic pressure.^[21]^

#### 2.2.3. Covariates

To control confounding, we extracted demographic, socioeconomic, clinical and lifestyle variables from NHANES: age, sex, race/ethnicity (Hispanic, non-Hispanic White, non-Hispanic Black, other), education (<9, 9–12, ≥12 years), poverty-income ratio (low ≤ 1.3, moderate 1.3–3.5, high > 3.5), physician-diagnosed coronary heart disease, diabetes or stroke, body-mass index (kg m^−2^) and smoking status (never: <100 cigarettes in lifetime; former: ≥100 cigarettes and quit; current: ≥100 cigarettes and currently smoking; based on NHANES definitions).We also included self-reported physical activity (total metabolic equivalent of task minutes per week, MET-min/week, continuous) and use of antihypertensive medications (yes/no).

#### 2.2.4. A statistical survey was conducted to obtain meaningful data

Baseline characteristics were compared with Student t tests (continuous variables) and χ^2^ tests (categorical variables). Subgroup heterogeneity was assessed by multivariable logistic regression, and the likelihood-ratio test evaluated interaction between B-vitamin intake (thiamin, riboflavin, niacin) and subgroups. All analyses were performed in R version 4.3.2 (R Foundation for Statistical Computing, Vienna, Austria).^[22]^

Categorical data are reported as unweighted counts (weighted percentages); continuous data as means ± standard errors. Group differences were tested with linear regression (continuous) and χ^2^ tests (categorical). Multivariable logistic regression estimated odds ratios (OR; 95% CI) for hypertension subtypes across quartiles of thiamin (B_1_), riboflavin (B_2_), and niacin (B_3_). Model 1: age, sex, race/ethnicity, education, poverty-income ratio (PIR). Model 2: Model 1 + diabetes, coronary heart disease, stroke. Model 3: Model 2 + body-mass index, smoking status, physical activity, and antihypertensive medication use. Sensitivity analyses repeated all models. Trend tests used quartile medians.

A 2-stage logistic regression model was established to evaluate the relationship between thiamin, riboflavin, and niacin and hypertension subtypes, with Model 3 including adjustments for potential confounding factors. Threshold (inflection point) analysis was performed for all 3 vitamins using a segmented logistic regression approach (Muggeo, 2003). Furthermore, to determine whether the relationships remained stable across different populations, interaction and subgroup analyses were conducted according to sex, ethnicity (Mexican American, non-Hispanic White, non-Hispanic Black, other), and BMI (<25, 25–30, >30 kg/m^2^). Heterogeneity and interactions across subgroups were assessed using logistic regression models and likelihood ratio tests, respectively. Statistical analyses were performed using R software version 4.2.1 (R Foundation for Statistical Computing, Vienna, Austria), the R survey package version 4.1-1, and FreeStat software version 1.7.1 (Beijing Free Clinical Medical Technology Co., Ltd.).^[23]^

## 3. Results

### 3.1. Study population

This real-time study ultimately included 17,484 participants from the National Health and Nutrition Examination Survey (NHANES) between 1999 and 2023. The detailed inclusion and exclusion process is illustrated in Figure 1.

### 3.2. Baseline characteristics

Table 1 summarizes baseline characteristics of 17,484 NHANES adults across quartiles of dietary thiamin, riboflavin, and niacin. Higher intake of each B-vitamin was associated with younger age, male predominance, and lower crude prevalence of isolated systolic hypertension and elevated pulse pressure (Q4 vs Q1: thiamin 11.8% vs 16.2%; riboflavin 11.5% vs 16.7%; niacin 11.1% vs 17.3%; all *P* < .001). Participants in the top quartiles also exhibited higher socioeconomic status and reduced rates of diabetes and stroke. The proportion of antihypertensive medication use was similar across quartiles.

**Table 1:** Population characteristics by quartiles of total thiamin, riboflavin, and niacin intake.

Variables	Thiamine Intake, mg/d						Riboflavin Intake, mg/d						Niacin Intake, mg/d					
	Total	Q1	Q2	Q3	Q4	*P*	Total	Q1	Q2	Q3	Q4	*P*	Total	Q1	Q2	Q3	Q4	*P*
		(≤0.965)	(0.966–1.399)	(1.400–1.974)	(≥1.975)			(≤1.294)	(1.295–1.887)	(1.888–2.630)	(≥2.631)			(≤14.584)	(14.585–21.376)	(21.377–30.844)	(≥30.845)	
NO.	17,484	4364	4374	4374	4372		17,484	4369	4369	4375	4371		17,484	4371	4371	4371	4371	
Isolated systolic hypertension, n (weighted %)						<.001						<.001						<.001
Yes	2521 (14.4)	706 (16.2)	677 (15.5)	622 (14.2)	516 (11.8)		2521 (14.4)	728 (16.7)	695 (15.9)	595 (13.6)	503 (11.5)		2521 (14.4)	757 (17.3)	654 (15)	627 (14.3)	483 (11.1)	
No	14,963 (85.6)	3658 (83.8)	3697 (84.5)	3752 (85.8)	3856 (88.2)		14,963 (85.6)	3641 (83.3)	3674 (84.1)	3780 (86.4)	3868 (88.5)		14,963 (85.6)	3614 (82.7)	3717 (85)	3744 (85.7)	3888 (88.9)	
Isolated diastolic hypertension, n (weighted %)						.265						.442						.231
Yes	350 (2.0)	86 (2)	75 (1.7)	101 (2.3)	88 (2)		350 (2.0)	81 (1.9)	100 (2.3)	82 (1.9)	87 (2)		350 (2.0)	95 (2.2)	71 (1.6)	92 (2.1)	92 (2.1)	
No	17,134 (98.0)	4278 (98)	4299 (98.3)	4273 (97.7)	4284 (98)		17,134 (98.0)	4288 (98.1)	4269 (97.7)	4293 (98.1)	4284 (98)		17,134 (98.0)	4276 (97.8)	4300 (98.4)	4279 (97.9)	4279 (97.9)	
Both hypertension, n (weighted %)						.002						< .001						.159
Yes	720 (4.1)	222 (5.1)	176 (4)	160 (3.7)	162 (3.7)		720 (4.1)	243 (5.6)	176 (4)	149 (3.4)	152 (3.5)		720 (4.1)	181 (4.1)	204 (4.7)	166 (3.8)	169 (3.9)	
No	16,764 (95.9)	4142 (94.9)	4198 (96)	4214 (96.3)	4210 (96.3)		16,764 (95.9)	4126 (94.4)	4193 (96)	4226 (96.6)	4219 (96.5)		16,764 (95.9)	4190 (95.9)	4167 (95.3)	4205 (96.2)	4202 (96.1)	
Pulse Pressure, n (weighted %)						<.001						<.001						<.001
Yes	5091 (29.1)	1351 (31)	1357 (31)	1288 (29.4)	1095 (25)		5091 (29.1)	1379 (31.6)	1361 (31.2)	1310 (29.9)	1041 (23.8)		5091 (29.1)	1413 (32.3)	1347 (30.8)	1269 (29)	1062 (24.3)	
No	12,393 (70.9)	3013 (69)	3017 (69)	3086 (70.6)	3277 (75)		12,393 (70.9)	2990 (68.4)	3008 (68.8)	3065 (70.1)	3330 (76.2)		12,393 (70.9)	2958 (67.7)	3024 (69.2)	3102 (71)	3309 (75.7)	
Age, Mean ± SD	53.3 ± 16.5	54.2 ± 16.3	54.5 ± 16.7	53.8 ± 16.6	50.6 ± 16.4	<.001	53.3 ± 16.5	53.5 ± 16.7	54.3 ± 16.7	54.2 ± 16.3	51.1 ± 16.3	<.001	53.3 ± 16.5	54.9 ± 16.6	54.9 ± 16.6	53.7 ± 16.3	49.7 ± 16.1	<.001
Gender, n (weighted %)						<.001						<.001						<.001
Male	9983 (57.1)	1804 (41.3)	2232 (51)	2641 (60.4)	3306 (75.6)		9983 (57.1)	1929 (44.2)	2228 (51)	2616 (59.8)	3210 (73.4)		9983 (57.1)	1772 (40.5)	2225 (50.9)	2698 (61.7)	3288 (75.2)	
Female	7501 (42.9)	2560 (58.7)	2142 (49)	1733 (39.6)	1066 (24.4)		7501 (42.9)	2440 (55.8)	2141 (49)	1759 (40.2)	1161 (26.6)		7501 (42.9)	2599 (59.5)	2146 (49.1)	1673 (38.3)	1083 (24.8)	
Race, n (weighted %)						<.001						<.001						<.001
Non-Hispanic white	9624 (55.0)	2225 (51)	2410 (55.1)	2480 (56.7)	2509 (57.4)		9624 (55.0)	1834 (42)	2339 (53.5)	2612 (59.7)	2839 (65)		9624 (55.0)	2270 (51.9)	2443 (55.9)	2476 (56.6)	2435 (55.7)	
Non-Hispanic black	3275 (18.7)	1045 (23.9)	844 (19.3)	725 (16.6)	661 (15.1)		3275 (18.7)	1267 (29)	873 (20)	616 (14.1)	519 (11.9)		3275 (18.7)	928 (21.2)	821 (18.8)	767 (17.5)	759 (17.4)	
Mexican American	2230 (12.8)	528 (12.1)	565 (12.9)	547 (12.5)	590 (13.5)		2230 (12.8)	585 (13.4)	581 (13.3)	564 (12.9)	500 (11.4)		2230 (12.8)	620 (14.2)	531 (12.1)	509 (11.6)	570 (13)	
Others	2355 (13.5)	566 (13)	555 (12.7)	622 (14.2)	612 (14)		2355 (13.5)	683 (15.6)	576 (13.2)	583 (13.3)	513 (11.7)		2355 (13.5)	553 (12.7)	576 (13.2)	619 (14.2)	607 (13.9)	
Education.Level, n (weighted %)						<.001						<.001						<.001
< 9	1611 (9.2)	482 (11)	422 (9.7)	357 (8.2)	350 (8)		1611 (9.2)	520 (11.9)	424 (9.7)	366 (8.4)	301 (6.9)		1611 (9.2)	579 (13.3)	432 (9.9)	321 (7.3)	279 (6.4)	
9–12	7525 (43.1)	2013 (46.1)	1871 (42.8)	1790 (40.9)	1851 (42.3)		7525 (43.1)	2074 (47.5)	1853 (42.4)	1773 (40.5)	1825 (41.8)		7525 (43.1)	1966 (45)	1865 (42.7)	1865 (42.7)	1829 (41.8)	
>12	8341 (47.7)	1867 (42.8)	2079 (47.6)	2225 (50.9)	2170 (49.6)		8341 (47.7)	1771 (40.6)	2091 (47.9)	2235 (51.1)	2244 (51.4)		8341 (47.7)	1821 (41.7)	2072 (47.4)	2185 (50)	2263 (51.8)	
Family PIR infmpir, n (weighted %)						<.001						<.001						<.001
Low	3659 (20.9)	1140 (26.1)	862 (19.7)	804 (18.4)	853 (19.5)		3659 (20.9)	1219 (27.9)	874 (20)	787 (18)	779 (17.8)		3659 (20.9)	1121 (25.6)	880 (20.1)	820 (18.8)	838 (19.2)	
Medium	7617 (43.6)	1954 (44.8)	1940 (44.4)	1869 (42.7)	1854 (42.4)		7617 (43.6)	1993 (45.6)	1945 (44.5)	1868 (42.7)	1811 (41.4)		7617 (43.6)	1979 (45.3)	1946 (44.5)	1832 (41.9)	1860 (42.6)	
High	6208 (35.5)	1270 (29.1)	1572 (35.9)	1701 (38.9)	1665 (38.1)		6208 (35.5)	1157 (26.5)	1550 (35.5)	1720 (39.3)	1781 (40.7)		6208 (35.5)	1271 (29.1)	1545 (35.3)	1719 (39.3)	1673 (38.3)	
Diabetes, n (weighted %)						<.001						<.001						<.001
Yes	2450 (14.0)	631 (14.5)	652 (14.9)	638 (14.6)	529 (12.1)		2450 (14.0)	662 (15.2)	664 (15.2)	640 (14.6)	484 (11.1)		2450 (14.0)	673 (15.4)	674 (15.4)	603 (13.8)	500 (11.4)	
No	15,025 (86.0)	3731 (85.5)	3720 (85.1)	3733 (85.4)	3841 (87.9)		15,025 (86.0)	3704 (84.8)	3703 (84.8)	3734 (85.4)	3884 (88.9)		15,025 (86.0)	3697 (84.6)	3694 (84.6)	3764 (86.2)	3870 (88.6)	
Coronary heart disease, n (weighted %)						.525						.074						<.001
Yes	1056 (6.0)	255 (5.8)	283 (6.5)	272 (6.2)	246 (5.6)		1056 (6.0)	277 (6.3)	256 (5.9)	290 (6.6)	233 (5.3)		1056 (6.0)	256 (5.9)	311 (7.1)	280 (6.4)	209 (4.8)	
No	16,350 (93.5)	4089 (93.7)	4067 (93)	4084 (93.4)	4110 (94)		16,350 (93.5)	4066 (93.1)	4095 (93.7)	4065 (92.9)	4124 (94.3)		16,350 (93.5)	4086 (93.5)	4038 (92.4)	4076 (93.3)	4150 (94.9)	
Stroke, n (weighted %)						<.001						<.001						<.001
Yes	865 (4.9)	274 (6.3)	229 (5.2)	198 (4.5)	164 (3.8)		865 (4.9)	294 (6.7)	209 (4.8)	208 (4.8)	154 (3.5)		865 (4.9)	289 (6.6)	234 (5.4)	202 (4.6)	140 (3.2)	
No	16,590 (94.9)	4084 (93.6)	4134 (94.5)	4169 (95.3)	4203 (96.1)		16,590 (94.9)	4067 (93.1)	4153 (95.1)	4160 (95.1)	4210 (96.3)		16,590 (94.9)	4074 (93.2)	4130 (94.5)	4160 (95.2)	4226 (96.7)	
SMOKE, n (weighted %)						<.001						<.001						<.001
Never	9645 (55.2)	2231 (51.1)	2519 (57.6)	2684 (61.4)	2211 (50.6)		9944 (56.9)	2262 (51.8)	2575 (58.9)	2638 (60.3)	2469 (56.5)		9840 (56.3)	2334 (53.4)	2584 (59.1)	2586 (59.2)	2336 (53.4)	
Former	4039 (23.1)	1022 (23.4)	1010 (23.1)	1016 (23.2)	991 (22.7)		4039 (23.1)	1027 (23.5)	1007 (23.0)	1015 (23.2)	990 (22.7)		3944 (22.6)	1037 (23.7)	986 (22.6)	985 (22.5)	936 (21.4)	
Current	3800 (21.7)	1111 (25.5)	845 (19.3)	674 (15.4)	1170 (26.8)		3501 (20.0)	1080 (24.7)	787 (18.0)	722 (16.5)	912 (20.9)		3700 (21.2)	1000 (22.9)	801 (18.3)	800 (18.3)	1099 (25.1)	
Body mass index (kg/m2), n (weighted %)						<.001						<.001						.001
< 25	4925 (28.2)	1208 (27.7)	1249 (28.6)	1159 (26.5)	1309 (29.9)		4925 (28.2)	1183 (27.1)	1210 (27.7)	1269 (29)	1263 (28.9)		4925 (28.2)	1183 (27.1)	1221 (27.9)	1237 (28.3)	1284 (29.4)	
25–30	5957 (34.1)	1419 (32.5)	1493 (34.1)	1516 (34.7)	1529 (35)		5957 (34.1)	1436 (32.9)	1438 (32.9)	1502 (34.3)	1581 (36.2)		5957 (34.1)	1447 (33.1)	1466 (33.5)	1495 (34.2)	1549 (35.4)	
>30	6602 (37.8)	1737 (39.8)	1632 (37.3)	1699 (38.8)	1534 (35.1)		6602 (37.8)	1750 (40.1)	1721 (39.4)	1604 (36.7)	1527 (34.9)		6602 (37.8)	1741 (39.8)	1684 (38.5)	1639 (37.5)	1538 (35.2)	
Supplement Use, n (weighted %)	5669 (32.4)	764 (17.5)	1124 (25.7)	1632 (37.3)	2149 (49.2)	<.001	5860 (33.5)	842 (19.3)	1255 (28.7)	1680 (38.4)	2083 (47.7)	<.001	5870 (33.6)	798 (18.3)	1280 (29.3)	1695 (38.8)	2097 (48.0)	<.001
Antihypertensive Medication Use, n (weighted %)	5120 (29.3)	1351 (31.0)	1357 (31.0)	1288 (29.4)	1124 (25.7)	<.001	5120 (29.3)	1379 (31.6)	1361 (31.2)	1310 (29.9)	1070 (24.5)	<.001	5120 (29.3)	1413 (32.3)	1347 (30.8)	1269 (29.0)	1091 (25.0)	<.001
Physical Activity, MET-min/wk, Mean ± SD	3025 ± 185	2450 ± 85	2850 ± 90	3200 ± 95	3650 ± 100	<.001	3025 ± 185	2480 ± 88	2870 ± 92	3180 ± 96	3580 ± 105	<.001	3025 ± 185	2420 ± 82	2860 ± 90	3220 ± 98	3610 ± 110	<.001

BMI = body mass index, CI = confidence interval, MET = metabolic equivalent of task, OR = odds ratio, PIR = poverty-income ratio.

### 3.3. Multivariable analysis

After full adjustment (Model 3), the top quartile of thiamin remained positively associated with hypertension (OR 1.17, 95% CI 1.04–1.31; *P*-trend = .009); riboflavin showed the steepest dose–response (OR 1.23, 1.10–1.39; *P*-trend < .001) and niacin a weaker signal (OR 1.16, 1.03–1.30; *P*-trend = .022), implying that high-dose B-vitamin intake may modestly elevate blood pressure (Table 2). In contrast, only riboflavin was inversely related to elevated pulse pressure (OR 0.84, 0.75–0.94; *P*-trend = .003), whereas thiamin and niacin were neutral (*P*-trend ≥ .24) (Table 3).These divergent associations suggest riboflavin selectively attenuates arterial stiffness, possibly via one-carbon–dependent pathways, rather than reflecting a generic B-vitamin effect.

**Table 2:** Association between total thiamin, riboflavin, and niacin intake (quartiles) and hypertension among adult participants in NHANES 1999–2023.

Quartiles	OR (95% CI)								
	No.	Crude	*P*-Value	Model 1	*P*-Value	Model 2	*P*-Value	Model 3	*P*-Value
**Thiamine**	17,484								
Thiamine cut1 (≤0.965)	4364	1 (Ref)		1 (Ref)		1 (Ref)		1 (Ref)	
Thiamine cut2 (0.966–1.399)	4374	1.12 (1.02~1.24)	.023	1.11 (1~1.23)	.06	1.11 (0.99~1.23)	.063	1.07 (0.97~1.18)	.071
Thiamine cut3 (1.400–1.974)	4374	1.2 (1.08~1.33)	.001	1.11 (1~1.24)	.06	1.11 (0.99~1.24)	.065	1.14 (1.03~1.26)	.048
Thiamine cut4 (≥1.975)	4372	1.42 (1.28~1.58)	<.001	1.17 (1.04~1.31)	.007	1.17 (1.04~1.31)	.009	1.17 (1.04~1.31)	.008
Trend.test			<.001		.01		.012		.009
**Riboflavin**	17,484								
Riboflavin cut1 (≤1.294)	4369	1 (Ref)		1 (Ref)		1 (Ref)		1 (Ref)	
Riboflavin cut2 (1.295–1.887)	4369	1.11 (1~1.23)	.04	1.05 (0.95~1.17)	.324	1.05 (0.95~1.17)	.326	1.10 (1~1.21)	.305
Riboflavin cut3 (1.888–2.630)	4375	1.36 (1.23~1.51)	<.001	1.24 (1.11~1.39)	<.001	1.24 (1.11~1.39)	<.001	1.19 (1.08~1.31)	<.001
Riboflavin cut4 (≥2.631)	4371	1.55 (1.4~1.72)	<.001	1.24 (1.1~1.39)	<.001	1.23 (1.1~1.39)	<.001	1.23 (1.1~1.39)	<.001
Trend.test			<.001		<.001		<.001		<.001
Niacin	17,484								
Niacin.cut1 (≤14.584)	4371	1 (Ref)		1 (Ref)		1 (Ref)		1 (Ref)	
Niacin.cut2(14.585–21.376)	4371	1.15 (1.04~1.27)	.008	1.11 (1~1.24)	.048	1.11 (1~1.24)	.052	1.05 (0.96~1.15)	.048
Niacin.cut3 (21.377–30.844)	4371	1.22 (1.1~1.35)	<.001	1.09 (0.98~1.22)	.107	1.09 (0.98~1.22)	.117	1.11 (1.01~1.22)	.109
Niacin.cut4(≥30.845)	4371	1.51 (1.36~1.68)	<.001	1.16 (1.04~1.31)	.01	1.16 (1.03~1.3)	.013	1.16 (1.03~1.3)	.013
Trend.test			<.001		.018		.023		.022

Model 1: Adjusted for age, sex, race/ethnicity, educational attainment, and household income ratio. Model 2: Model 1 + history of diabetes, coronary heart disease, and stroke. Model 3: Model 2 + body mass index, smoking status, physical activity, and antihypertensive medication use.

**Table 3:** Association between total thiamin, riboflavin, and niacin intake (quartiles) and increased pulse pressure in adult participants of NHANES 1999–2023.

Quartiles	OR (95% CI)								
	No.	Crude	*P*-Value	Model 1	*P*-Value	Model 2	*P*-Value	Model 3	*P*-Value
**Thiamine**	17,484								
Thiamine cut1 (≤0.965)	4364	1 (Ref)		1 (Ref)		1 (Ref)		1 (Ref)	
Thiamine cut2 (0.966–1.399)	4374	1 (0.92~1.1)	.946	1.01 (0.91~1.12)	.853	1 (0.91~1.11)	.935	0.99 (0.89~1.10)	.99
Thiamine cut3 (1.400–1.974)	4374	0.93 (0.85~1.02)	.124	1 (0.91~1.11)	.945	1 (0.9~1.11)	.98	0.98 (0.88~1.09)	0.9
Thiamine cut4 (≥1.975)	4372	0.75 (0.68~0.82)	<.001	0.94 (0.84~1.04)	.237	0.94 (0.84~1.05)	.262	0.96 (0.86~1.07)	.218
Trend.test			<.001		.266		.289		.24
**Riboflavin**	17,484								
Riboflavin cut1 (≤1.294)	4369	1 (Ref)		1 (Ref)		1 (Ref)		1 (Ref)	
Riboflavin cut2 (1.295–1.887)	4369	0.98 (0.9~1.07)	.678	1.01 (0.91~1.11)	.906	1 (0.91~1.11)	.935	0.94 (0.85~1.04)	.968
Riboflavin cut3 (1.888–2.630)	4375	0.93 (0.85~1.01)	.101	0.99 (0.89~1.1)	.846	0.99 (0.89~1.09)	.796	0.90 (0.81~1.00)	.759
Riboflavin cut4 (≥2.631)	4371	0.68 (0.62~0.74)	<.001	0.83 (0.75~0.93)	.001	0.84 (0.75~0.94)	.002	0.84 (0.75~0.94)	.002
Trend.test			<.001		.002		.003		.003
Niacin	17,484								
Niacin.cut1 (≤14.584)	4371	1 (Ref)		1 (Ref)		1 (Ref)		1 (Ref)	
Niacin.cut2(14.585–21.376)	4371	0.93 (0.85~1.02)	.129	0.96 (0.87~1.06)	.464	0.96 (0.87~1.06)	.41	0.99 (0.90~1.09)	.374
Niacin.cut3 (21.377–30.844)	4371	0.86 (0.78~0.94)	.001	0.98 (0.88~1.08)	.65	0.98 (0.88~1.08)	.642	0.97 (0.88~1.07)	.59
Niacin.cut4(≥30.845)	4371	0.67 (0.61~0.74)	<.001	0.95 (0.85~1.05)	.315	0.95 (0.86~1.06)	.394	0.96 (0.86~1.07)	.35
Trend.test			<.001		.385		.479		.429

Model 1: Adjusted for age, sex, race/ethnicity, educational attainment, and household income ratio. Model 2: Model 1 + history of diabetes, coronary heart disease, and stroke. Model 3: Model 2 + body mass index, smoking status, physical activity, and antihypertensive medication use.

### 3.4. nonlinear relationship

**Figure 2.**
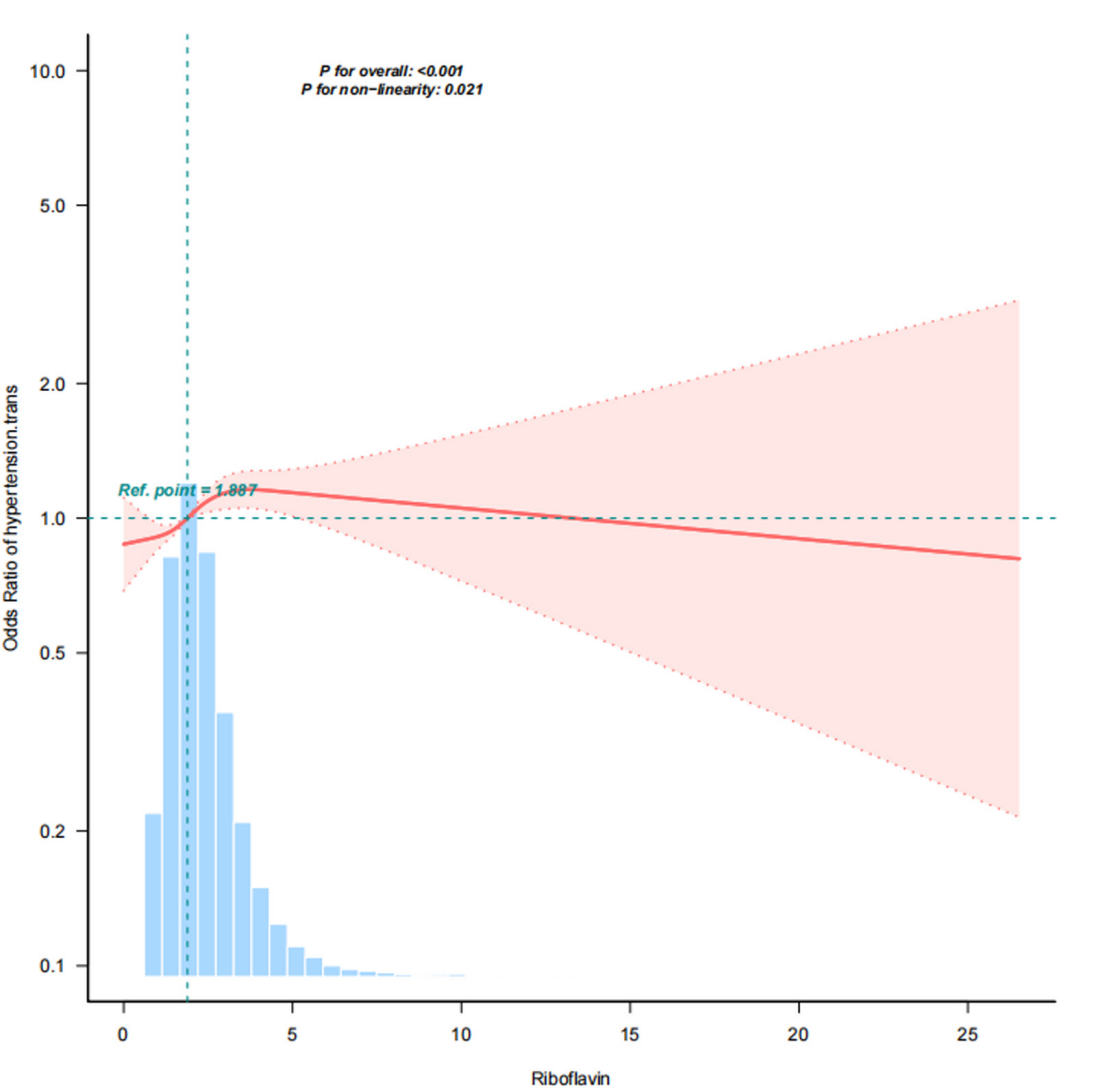
Nonlinear relationship between riboflavin intake and hypertension risk. The solid line represents the adjusted odds ratio, and the shaded area represents the 95% confidence interval. Models were adjusted for all covariates in Model 3.

**Figure 3.**
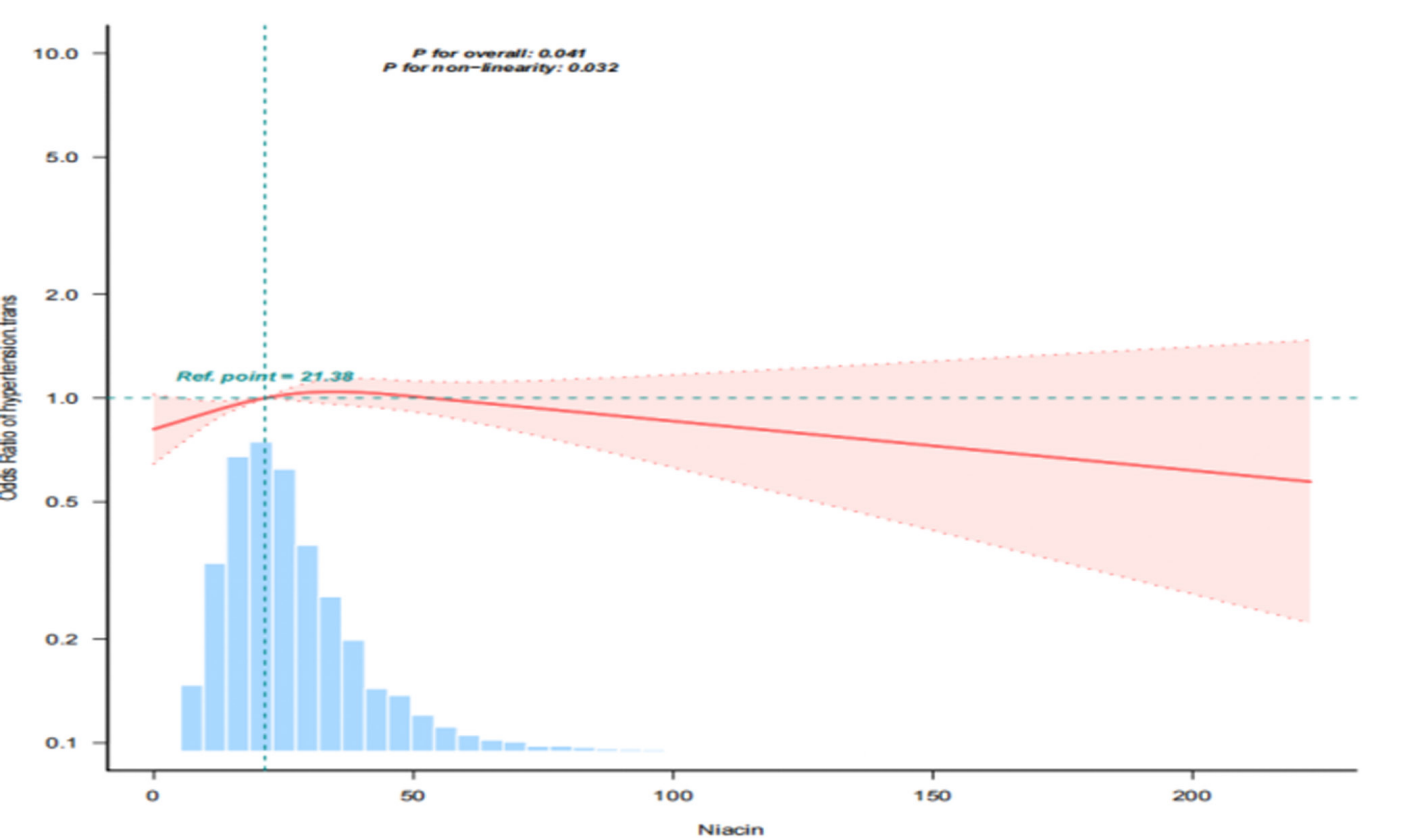
Nonlinear relationship between niacin intake and hypertension risk. The solid line represents the adjusted odds ratio, and the shaded area represents the 95% confidence interval. Models were adjusted for all covariates in Model 3.

Figure 2 demonstrates a nonlinear, inverse dose–response relationship between riboflavin intake and hypertension incidence (overall *P* < .001; nonlinear *P* = .021). Risk declined sharply at approximately 2 mg/day intake, leveled off within the 2–5 mg/day range, and subsequently remained stable, indicating a threshold effect rather than sustained benefit. These data suggest that moderate riboflavin supplementation achieves vascular protection, with no additional advantage from higher doses. Figure 3 reveals a J-shaped nonlinear relationship between dietary niacin intake and hypertension risk (overall *P* = .032; nonlinear *P* = .041). Risk decreased marginally at approximately 20 mg/day intake, reached a minimum around 30–40 mg, and then gradually increased, indicating a potential U-shaped threshold. These findings suggest that optimal niacin intake within recommended ranges may confer vascular protective benefits, whereas excessive exposure may diminish or reverse this protective effect. Table 4 reveals a threshold-specific association between niacin intake and hypertension. Below 46.163 mg/day, each unit increase in intake showed a marginal association with elevated risk (OR 1.007; 95% CI 1.002–1.012; *P* = .0045), whereas the association leveled off beyond this threshold (OR 0.99; 95% CI 0.973–1.007; *P* = .26). Log-likelihood ratio tests failed to confirm a statistically significant turning point (*P* = .087). No significant threshold effects were detected for thiamin or riboflavin.

**Table 4:** Threshold effect analysis for the association between niacin intake and hypertension.

Niacin Intake (mg/d)	Adjusted Model	
	OR (95% CI)	*P* value
<**46.163**	1.007 (1.002~1.012)	.0045
≥46.163	0.99 (0.973~1.007)	.2625
Log-likelihood ratio test	-	.087

This table presents threshold effect analyses for all 3 vitamins. Only niacin showed a suggestive inflection point at 46.163 mg/d (P for LLR = 0.087). For thiamine and riboflavin, no significant threshold effects were detected (P for LLR > 0.05). Models were adjusted for all covariates in Model 3.

### 3.5. Subgroup analysis

**Figure 4.**
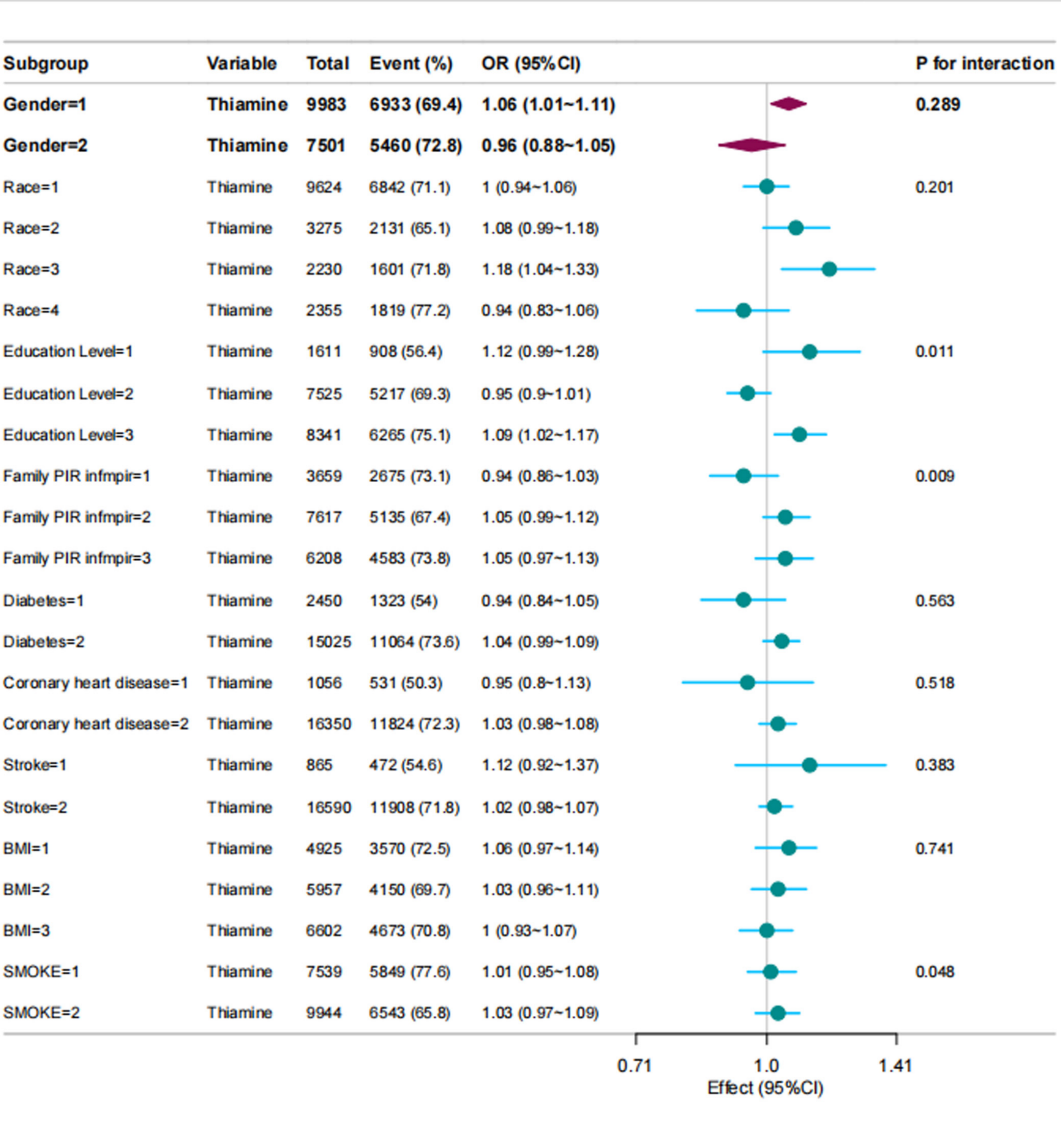
Forest plot for the association between total thiamin intake and hypertension across subgroups. ORs and 95% CIs are derived from fully adjusted logistic regression models within each stratum. P for interaction from likelihood ratio test. BMI = body mass index (kg/m^2^).

**Figure 5.**
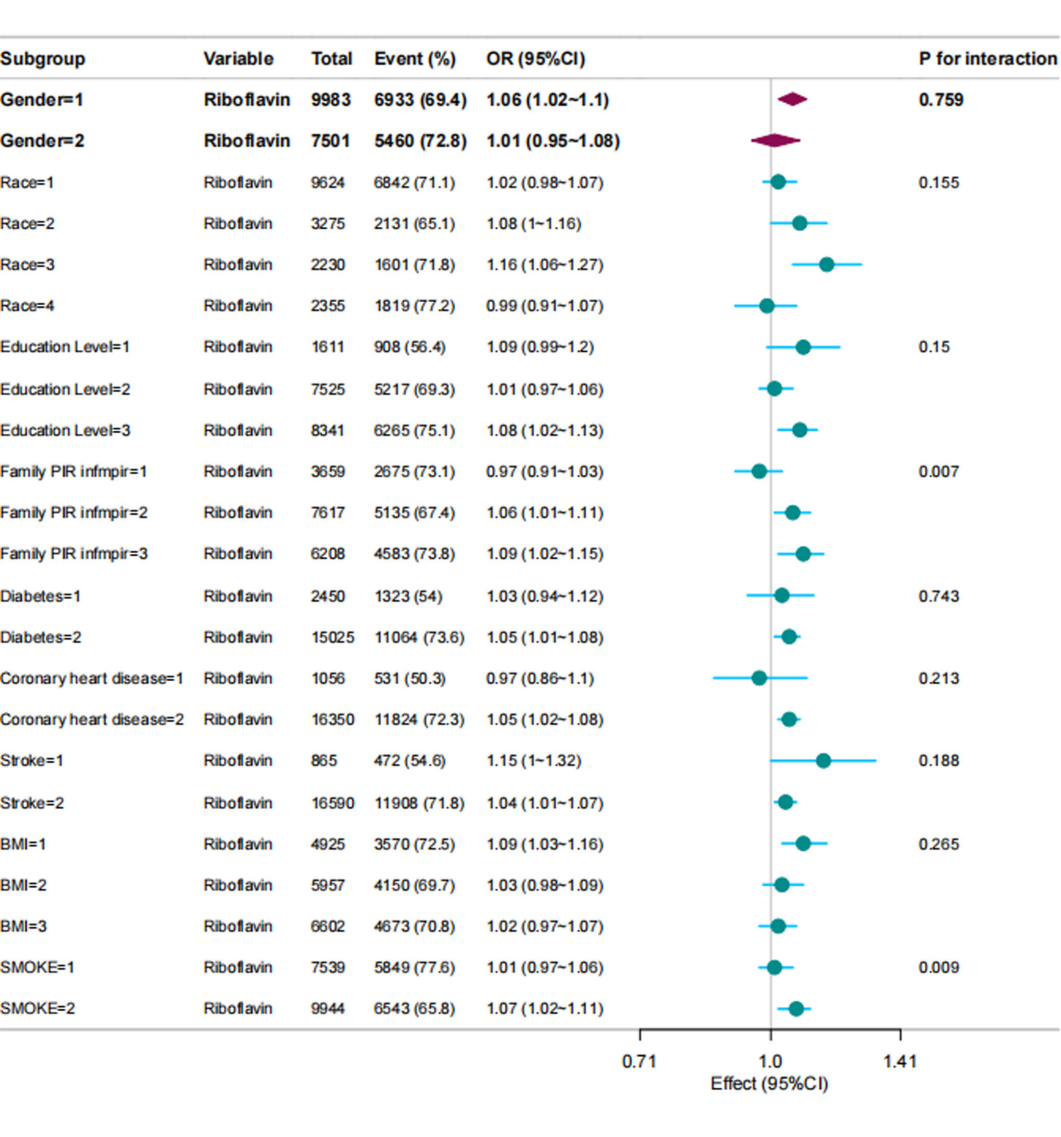
Forest plot for the association between total riboflavin intake and hypertension across subgroups. BMI = body mass index (kg/m^2^).

**Figure 6.**
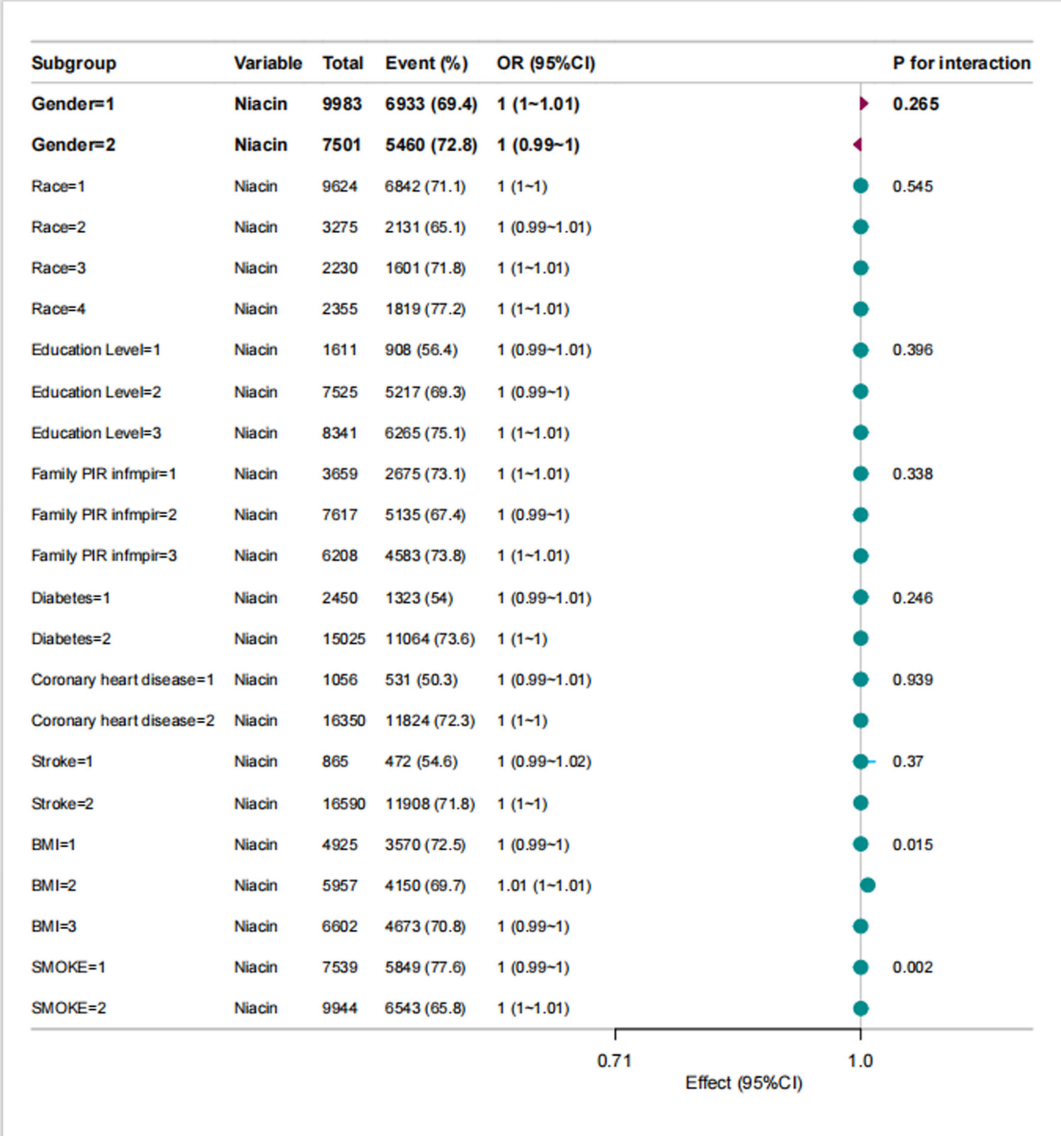
Forest plot for the association between total niacin intake and hypertension across subgroups. BMI = body mass index (kg/m^2^).

Figure 4 illustrates the association between dietary thiamin and hypertension. Overall, thiamin exhibits a modest positive correlation trend with no statistical heterogeneity (most stratified interaction *P*-values ≥ .05). Significant differences emerged only in the education level (*P* = .011) and income level (*P* = .009) subgroups – higher intake slightly increased risk among the most educated and highest-income individuals. Conversely, gender, ethnicity, BMI, diabetes, coronary heart disease, stroke, and smoking status consistently showed zero or negligible effects, indicating that the association between thiamin and hypertension is largely independent of conventional demographic and clinical factors. Figure 5 demonstrates a weak association between riboflavin intake and hypertension, consistent across all strata. Pooled estimates remained around 1.05, with only income (*P* = .007) and smoking (*P* = .009) showing minor interactions: higher riboflavin intake slightly increased risk only among high-income individuals and former smokers. Gender, ethnicity, educational attainment, BMI, and comorbidities all exhibited consistent null trends, indicating that riboflavin pressor effect was not substantially altered by conventional demographic or clinical factors. Figure 6 demonstrates that across all subgroups, niacin intake exhibited no association with hypertension, with pooled odds ratios predominantly clustered around 1.00 (95% CI 0.99–1.01). Formal tests for interaction involving factors such as gender, ethnicity, educational attainment, income, diabetes, coronary heart disease, and stroke yielded no statistically significant findings (all *P* ≥ .15). Only BMI (*P* = .015) and smoking (*P* = .002) demonstrated nominal heterogeneity, yet the effect sizes remained negligible. Overall, within the range of niacin exposure studied, the risk of hypertension was not substantially influenced by demographic or clinical factors.

## 4. Discussion

In this large, cross-sectional study of U.S. adults, we found that higher total intake (from both diet and supplements) of thiamin, riboflavin, and niacin was associated with increased odds of hypertension after comprehensive adjustment for confounders. Conversely, higher riboflavin intake was associated with lower odds of increased pulse pressure. We observed a J-shaped nonlinear relationship for niacin, with the lowest hypertension risk at moderate intake levels (30–40 mg/day), and a suggestive inflection point around 46 mg/day. These associations were largely consistent across key demographic and clinical subgroups.

Thiamine has a relatively short retention time in the body, lasting only approximately 1 to 3 weeks, and cannot be synthesized by the human body itself.^[24,25]^ Thiamine plays a crucial role in oxidative stress, energy metabolism, endothelial function, and antiinflammatory processes.^[26–28]^ Extensive literature demonstrates that thiamin deficiency elevates the risk of multiple systemic diseases.^[29]^ Thiamine may improve or reverse angina pectoris, myocardial infarction, cardiovascular disease, diabetes mellitus, dyslipidemia, obesity, and psychiatric disorders.^[30,31]^ Consuming a measured amount of thiamin may improve cardiac function,^[32]^ reduce systemic vascular resistance,^[33]^ and improve hemodynamic characteristics.^[34]^ Vitamin B2 is also known as riboflavin. Riboflavin is an essential water-soluble vitamin that functions as a coenzyme in energy metabolism and redox reactions, playing a role in carbon metabolism. Riboflavin may exert its effects through antiinflammatory and antioxidant capabilities,^[35]^ as well as through enhancing nitric oxide availability and endothelial function, whilst restoring MTHFR activity in vascular cells to exert a hypotensive effect. Furthermore, riboflavin has been reported as a primary determinant of plasma total homocysteine (tHcy) levels, with riboflavin supplementation significantly reducing tHcy concentrations. Elevated tHcy directly promotes angiotensin-converting enzyme activity, potentially leading to increased angiotensin II levels and subsequent hypertension. These findings suggest that tHcy reduction may represent an alternative pathway through which riboflavin exerts its blood pressure-lowering effects.^[36]^ Research indicates that in China, substantial intake of riboflavin may lower blood pressure.^[37]^ Niacin is commonly referred to as vitamin B3. The effect of niacin on blood pressure remains a subject of debate. Research suggests that niacin may lower blood pressure by altering cytoplasmic sodium concentrations. Research by Kelly et al indicates that nicotinic acid does not significantly reduce mean blood pressure within the short term (within 24 hours).^[38]^ A multicenter cohort study conducted among Chinese adults suggests a curvilinear relationship between niacin intake and the incidence of hypertension, with the lowest risk observed at daily intakes ranging from 14.3 to 16.7 mg.^[39]^ Previous studies have indicated that nicotinic acid may be associated with reducing the risk of cardiovascular diseases such as hypertension, coronary heart disease, and stroke, as well as improving outcomes.^[40,41]^

Our findings of positive associations between high B-vitamin intake and hypertension appear to contrast with some previous studies that reported protective or null effects. Several factors may explain this discrepancy. First, most prior studies assessed dietary intake only, whereas we examined total intake, including supplements. Supplement users may differ in health behaviors, socioeconomic status, and underlying health conditions, leading to residual confounding. Indeed, in our study, participants in the highest intake quartiles were younger, more likely male, more educated, and had fewer chronic conditions – a generally healthier profile that might otherwise predict lower hypertension risk. The positive associations observed after adjustment suggest that high vitamin intake itself, particularly from supplements, might have a modest adverse effect on blood pressure, or that residual confounding (e.g., by other dietary components like sodium or unmeasured lifestyle factors) persists. Second, the U.S. population has a high prevalence of fortification and supplement use, leading to intake levels that may exceed those in populations studied previously. The potential for adverse effects at very high doses, as suggested by our niacin findings, warrants consideration. Third, differences in study design (cross-sectional vs prospective) and adjustment for confounders (e.g., physical activity, medication use) could influence the results.

The inverse association between riboflavin and pulse pressure is novel and biologically plausible. Pulse pressure is a marker of arterial stiffness. Riboflavin, as a precursor for FAD, is essential for the activity of methylenetetrahydrofolate reductase (MTHFR), a key enzyme in homocysteine metabolism. Riboflavin supplementation has been shown to lower homocysteine, particularly in individuals with the MTHFR 677TT genotype, and improved endothelial function could reduce arterial stiffness.^[36,37]^ However, our study did not measure homocysteine or arterial stiffness directly, so this mechanism remains speculative.

The J-shaped relationship for niacin aligns with the principle of nutrient “U-toxicity,” where both deficiency and excess are detrimental. Niacin vasodilatory effects at moderate doses, mediated through prostaglandin D2 release, could be beneficial, while very high doses, often from supplements, might induce pro-inflammatory responses or adverse metabolic effects that counteract any benefit.^[39,41]^ The nonsignificant threshold test (*P* = .087) indicates this finding is suggestive and requires replication.

## 5. Limitations

This study has several limitations. First, the cross-sectional design precludes causal inference; reverse causality (e.g., individuals with hypertension changing their diet or taking supplements) is possible. Second, although we adjusted for many confounders, residual confounding by unmeasured or imprecisely measured factors (e.g., detailed dietary patterns, sodium and potassium intake, stress, sleep quality, and duration of hypertension) cannot be ruled out. The lack of adjustment for sodium intake is a particular limitation. Third, vitamin intake was assessed primarily via a single 24-hour dietary recall, which may not reflect habitual intake and is subject to measurement error. However, supplement use was assessed over 30 days, improving accuracy for that component. Fourth, blood pressure was measured at a single visit, which may not capture true hypertensive status. However, NHANES uses a rigorous protocol. Fifth, we cannot separate the effects of dietary vs supplemental sources of vitamins in the main analysis due to collinearity and modeling constraints. Future studies should explore this distinction.

## 6. Conclusions

In In this nationally representative sample of U.S. adults, higher total intakes of thiamin, riboflavin, and niacin were associated with greater odds of hypertension, while higher riboflavin intake was associated with lower odds of increased pulse pressure. Niacin intake exhibited a J-shaped association with hypertension. These findings suggest that the relationship between B-vitamin intake and blood pressure is complex and may not be uniformly beneficial at high intake levels, particularly when derived from supplements. They underscore the importance of considering total nutrient intake from all sources in dietary guidance. Further prospective and intervention studies are needed to confirm these associations, elucidate the underlying mechanisms, and determine optimal intake ranges for cardiovascular health.

## Acknowledgments

The authors acknowledge Jie Liu of the Department of Vascular and Endovascular Surgery, Chinese PLA General Hospital, for his contribution to statistical support, study design consultations, and comments regarding the manuscript. We would like to thank all participants in this study.

## Author contributions

**Conceptualization:** Siqi Ma.

**Methodology:** Siqi Ma.

**Writing – original draft:** Siqi Ma.

**Data curation:** Qu Jin, Keying Yu, Hezeng Dong, Yanmei Lu.

**Formal analysis:** Qu Jin, Keying Yu, Hezeng Dong, Yanmei Lu.

**Funding acquisition:** Liping Chang.

**Supervision:** Liping Chang.

**Writing – review & editing:** Liping Chang.
